# Environmentally-triggered contraction of the norovirus virion determines diarrheagenic potential

**DOI:** 10.3389/fimmu.2022.1043746

**Published:** 2022-11-01

**Authors:** Emily W. Helm, Amy M. Peiper, Matthew Phillips, Caroline G. Williams, Michael B. Sherman, Theresa Kelley, Hong Q. Smith, Sorin O. Jacobs, Dhairya Shah, Sarah M. Tatum, Neha Iyer, Marco Grodzki, Joyce C. Morales Aparicio, Elizabeth A. Kennedy, Mikayla S. Manzi, Megan T. Baldridge, Thomas J. Smith, Stephanie M. Karst

**Affiliations:** ^1^ Department of Molecular Genetics & Microbiology, College of Medicine, University of Florida, Gainesville, FL, United States; ^2^ Department of Biochemistry and Molecular Biology, University of Texas Medical Branch at Galveston, Galveston, TX, United States; ^3^ Division of Infectious Diseases, Department of Medicine, Edison Family Center for Genome Sciences and Systems Biology, Washington University School of Medicine, St. Louis, MO, United States

**Keywords:** norovirus, gastrointestinal infections, calicivirus, infectious diseases, animal model

## Abstract

Noroviruses are the leading cause of severe childhood diarrhea and foodborne disease worldwide. While they are a major cause of disease in all age groups, infections in the very young can be quite severe with annual estimates of 50,000-200,000 fatalities in children under 5 years old. In spite of the remarkable disease burden associated with norovirus infections in people, very little is known about the pathogenic mechanisms underlying norovirus diarrhea, principally because of the lack of tractable small animal models. We recently demonstrated that wild-type neonatal mice are susceptible to murine norovirus (MNV)-induced acute self-resolving diarrhea in a time course mirroring human norovirus disease. Using this robust pathogenesis model system, we demonstrate that virulence is regulated by the responsiveness of the viral capsid to environmental cues that trigger contraction of the VP1 protruding (P) domain onto the particle shell, thus enhancing receptor binding and infectivity. The capacity of a given MNV strain to undergo this contraction positively correlates with infection of cells expressing low abundance of the virus receptor CD300lf, supporting a model whereby virion contraction triggers infection of CD300lf^lo^ cell types that are responsible for diarrhea induction. These findings directly link environmentally-influenced biophysical features with norovirus disease severity.

## Introduction

Human noroviruses are the leading cause of severe childhood diarrhea and gastroenteritis outbreaks globally, responsible for an estimated 685 million total cases and 50,000-200,000 deaths in young children each year (1–4). Understanding the pathogenesis of noroviruses, particularly in young hosts, is thus an important priority to aid in the development of vaccines and therapeutics. Yet very little is known about the mechanisms underlying human norovirus disease due to a lack of robust symptomatic *in vivo* model systems. While the murine norovirus (MNV) model system has facilitated investigation of certain aspects of norovirus pathogenesis over the past two decades, its utility has been limited because wild-type adult mice do not develop overt disease. We have recently demonstrated that wild-type neonatal mice develop acute self-resolving diarrhea when infected with MNV (5, 6), providing the opportunity to elucidate underlying mechanisms of intestinal disease in a tractable small animal model.

One of the strengths of the MNV model system is the availability of genotypically similar but phenotypically distinct virus strains, enabling identification of viral determinants of phenotypes of interest. For example, certain MNV strains (e.g., MNV1) infect intestinal immune cells and are cleared acutely from adult mice whereas others (e.g., CR6) establish persistent infection in colonic tuft cells (7–9). This differential cellular tropism is remarkable considering that all MNV strains characterized to date are highly genetically related intra-cluster strains and rely on the same host receptor, CD300lf, which is expressed on both immune and tuft cells (10–13). The ability of CR6 to establish tuft cell persistence in adult mice has been linked to the type III interferon (IFN)-antagonizing activity of the viral nonstructural protein NS1 (14–16). However, it remains unresolved why MNV1 is able to infect immune cells *in vivo* whereas CR6 infection is limited to tuft cells. This virus strain variability in cellular tropism may extend to human noroviruses since both epithelial and immune cell infection have been reported in various animal model and human biopsy studies (17–22). Moreover, human norovirus strains display much greater genetic heterogeneity than do MNV strains, raising the possibility that there are even more stark phenotypic distinctions among them. Importantly, immune cell-tropic MNV1 is more virulent than tuft cell-tropic CR6 in IFN-deficient adult mice and wild-type neonatal mice (5, 6, 23) so understanding the determinants of immune cell infection is key to elucidating how noroviruses cause disease. Herein we report that the protruding (P) domain of the major norovirus capsid protein VP1 is a key virulence determinant. VP1-associated virulence derives from the capacity of the P domain to undergo environmentally triggered contraction, enabling infection of receptor-low immune cell types. A recent study reveals that human noroviruses undergo a similar environmentally triggered contraction as MNV (24), demonstrating that this is a common feature of norovirus pathogenesis.

## Results

### The MNV1 VP1 capsid protein is a critical determinant of diarrhea in BALB/c neonates

**Figure 1:**
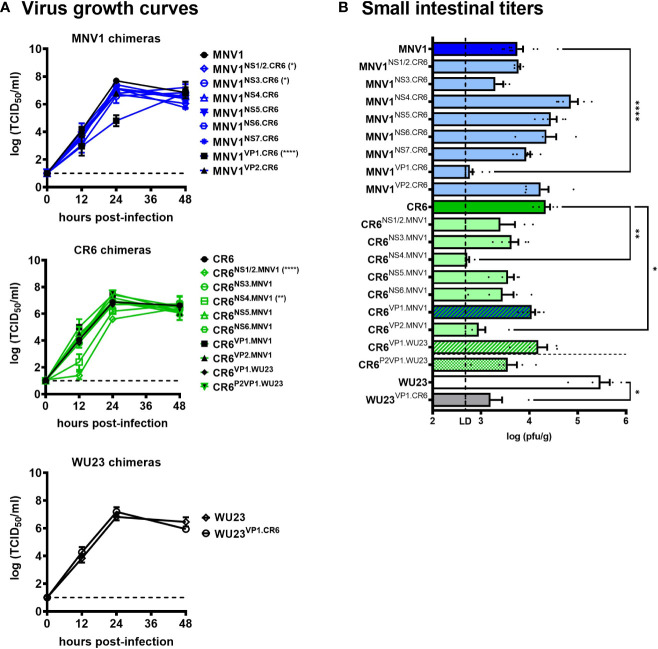
Replication efficiency of viral chimeras. **(A)** Duplicate wells of BV2 cells were infected with MNV1, CR6, WU23 or the indicated chimeric virus at MOI 0.05. Virus titers were determined by TCID_50_ assay on culture supernatants. Three independent experiments were performed. *P* values were determined using ANOVA with Dunnett’s multiple comparisons test by comparing each chimeric virus to its respective parental virus. **(B)** Groups of P3 BALB/c neonatal mice were inoculated with 10^7^ TCID_50_ units of MNV1, CR6, WU23 or the indicated chimeric virus. At 2d, viral titers were determined in the distal small intestine by plaque assay. At least five mice from at least two independent litters per condition were analyzed. *P* values were determined using ANOVA with Sidak’s multiple comparisons test by comparing each chimeric virus to its respective parental virus. *P < 0.05, **P < 0.01, ****P < 0.0001.

We have previously reported varying levels of virulence among commonly studied MNV strains in neonatal mice, with MNV1 and WU23 causing severe diarrhea and CR6 and MNV3 being attenuated (5, 6). Using an existing set of chimeric viruses in which single genes were reciprocally swapped between diarrheagenic MNV1 and attenuated CR6 (23), we performed a viral genome-wide analysis for virulence determinants. First, all chimeras were tested for their ability to replicate *in vitro* by performing viral growth curves in BV2 cells **(** **Figure 1A** **)** and *in vivo* by measuring virus titers in the small intestines of P3 BALB/c neonatal mice **(** **Figure 1B** **)**: On the MNV1 backbone, MNV1^NS1/2.CR6^ displayed a modest replication defect *in vitro* but it reached small intestinal titers comparable to parental MNV1 *in vivo*; MNV1^NS3.CR6^ displayed a modest replication defect *in vitro* and *in vivo*; while MNV1^VP1.CR6^ was substantially replication-impaired *in vitro* and *in vivo*. On the CR6 backbone, we were unable to recover CR6^NS7.MNV1^. Of the remaining seven CR6 chimeras, CR6^NS1/2.MNV1^ was replication-impaired *in vitro* although it reached small intestinal titers comparable to parental CR6 *in vivo*; CR6^NS4.MNV1^ was replication-impaired *in vitro* and *in vivo*; and CR6^VP2.MNV1^ was replication-impaired only *in vivo*. Based on their pronounced replication impairment, MNV1^VP1.CR6^, CR6^NS4.MNV1^, and CR6^VP2.MNV1^ were excluded from virulence studies.

**Figure 2:**
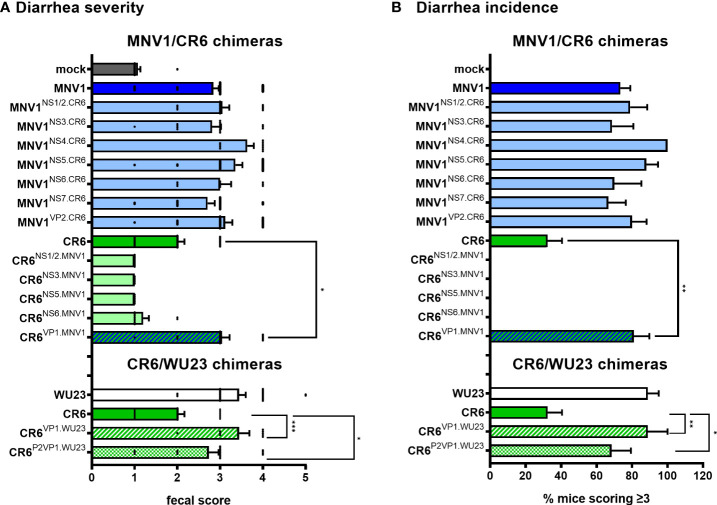
The surface-exposed domain of the major capsid protein VP1 is the major MNV virulence factor in BALB/c mice. **(A, B)** Groups of P3 BALB/c neonatal mice were inoculated with 10^7^ TCID_50_ units of mock inoculum, WU23, MNV1, CR6, or the indicated chimeric virus. **(A)** At 2 dpi, fecal consistency was determined by palpating their abdomens. **(B)** The proportion of mice scoring a 3 or above is presented as incidence of diarrhea. At least 5 mice from at least 2 independent litters per condition were analyzed. *P* values were determined for the MNV1/CR6 chimeras and the CR6/WU23 chimeras using Kruskal-Wallis test with Dunn’s multiple comparisons test by comparing each chimeric virus to its respective parental virus. *P < 0.05, **P < 0.01, ***P < 0.001.

To identify the viral factors responsible for the virulence difference between MNV1 and CR6, P3 BALB/c mice were infected with 10^7^ TCID_50_ units of each virus chimera and assessed for diarrhea at 2 dpi. All of the chimeras on the MNV1 backbone were as virulent as parental MNV1, while all of the chimeras on the CR6 backbone were as attenuated as parental CR6, with the exception of CR6^VP1.MNV1^ which caused more severe diarrhea than its parental counterpart **(** **Figure 2A** **)**. In addition to scoring fecal material for consistency, we also scored colon contents. These two measures of disease were highly concordant **(** **Supplemental Figure 1** **)**, providing increased confidence that CR6^VP1.MNV1^ was significantly more virulent than parental CR6. Of note, CR6^VP1.MNV1^ was as virulent as parental MNV1, causing diarrhea in 81% of pups compared to 73% of MNV1-infected pups and only 32% of CR6-infected pups **(** **Figure 2B** **)**, demonstrating that ORF2 encoding VP1 is fully responsible for the virulence difference between these two viruses. The VP1 protein, and specifically amino acid 296, has previously been implicated in virulence in adult IFN-deficient mice (23, 25, 26). All three prior studies on the role of this residue in virulence used lethality and weight loss as nonspecific measures of disease so the contribution of VP1 residue 296 to intestinal disease remains untested. In fact, this residue had no effect on diarrhea or colon content inconsistency in neonatal mice **(** **Supplemental Figure 2** **)**, underscoring the importance of studying pathogenesis in a model system recapitulating disease outcomes observed in human norovirus-infected people.

### VP1 is a virulence determinant across diarrheagenic MNV strainsand host strains

**Figure 3:**
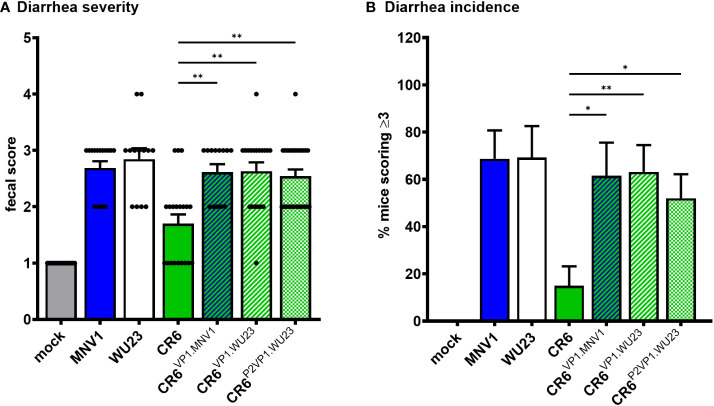
VP1 is the major MNV virulence factor in C57BL/6J mice. Groups of P3 B6 pups were inoculated with 10^7^ TCID_50_ units of mock inoculum, MNV1, WU23, CR6, or the indicated chimeric virus. **(A)** Fecal consistency was determined at 2 dpi by palpating their abdomens. **(B)** The proportion of mice scoring a 3 or above is presented as incidence of diarrhea. At least thirteen mice from a minimum of two different litters per condition were analyzed. Error bars denote standard errors of mean in all figures. *P* values were determined using Kruskal-Wallis test with Dunn’s multiple comparisons test comparing all groups to CR6. *P < 0.05, **P < 0.01.

Because the MNV strain WU23 is also diarrheagenic in neonatal mice (6), we questioned whether it shares virulence determinants with MNV1. While WU23^VP1.CR6^ replicated comparably to parental WU23 in BV2 cells **(** **Figure 1A** **)**, it was severely replication-impaired *in vivo* **(** **Figure 1B** **)**, confounding virulence studies. On the other hand, CR6^VP1.WU23^ was replication-competent *in vitro* and *in vivo* **(** **Figure 1** **)** so we tested its ability to cause intestinal disease in P3 BALB/c mice. Like CR6^VP1.MNV1^, CR6^VP1.WU23^ caused diarrhea **(** **Figure 2** **)** and colon content inconsistency **(** **Supplemental Figure 1** **)** that was comparably severe and penetrant as parental WU23, confirming that VP1 is singularly responsible for the virulence difference between diarrheagenic MNV strains like MNV1 and WU23 and attenuated CR6. We next sought to elucidate the domain of VP1 associated with diarrhea. The VP1 protein is comprised of a conserved shell (S) domain and variable protruding (P) domain, with the P domain further subdivided into the P1 stalk and hypervariable P2 tips of arches that extend off the surface of the virion (27, 28). As noted above, the P2 domain including residue 296 has previously been implicated in virulence in IFN-deficient mice (23, 25, 26). While residue 296 was not associated with diarrhea in neonates **(** **Supplemental Figure 2** **)**, it was still of interest to determine whether the P2 domain regulates diarrhea induction since it contains the CD300lf receptor binding pocket (29). CR6^P2VP1.WU23^ was replication-competent **(** **Figure 1** **)** and it caused more severe diarrhea than CR6 **(** **Figure 2** **)**. It should be noted that CR6^P2VP1.WU23^ was not as virulent as parental WU23 or CR6^VP1.WU23^ (e.g., it caused diarrhea in 68% of infected pups compared to 89% of WU23-infected pups and 89% of CR6^VP1.WU23^-infected pups) so residues within the S or P1 domains of VP1 may contribute to virulence cooperatively with residues in the P2 domain. Alternatively, this could reflect a slight reduction in infection efficiency of CR6^P2VP1.WU23^ considering that there was a modest, although not statistically significant, reduction in small intestinal titers in mice infected with this virus **(** **Figure 1B** **)**. Both BALB/c and C57BL/6J neonates are susceptible to MNV-induced diarrhea (5, 6). To confirm that similar virulence mechanisms operate in these two commonly studied mouse strains, P3 C57BL/6J mice were infected with 10^7^ TCID_50_ units of MNV1, WU23, CR6, CR6^VP1.MNV1^, CR6^VP1.WU23^, or CR6^P2VP1.WU23^. As observed in BALB/c pups, the full VP1 coding region from either diarrheagenic virus and the WU23 P2 domain conferred increased virulence to parental CR6 in C57BL/6J pups **(** **Figure 3** **)**. Overall, these results demonstrate that VP1 is a key virulence determinant for multiple diarrheagenic MNV strains and that virulence is controlled by the hypervariable surface-exposed P2 domain.

### MNV diarrheagenic potential correlates with the ability to infect CD300lf^lo^ immune cells

**Figure 4:**
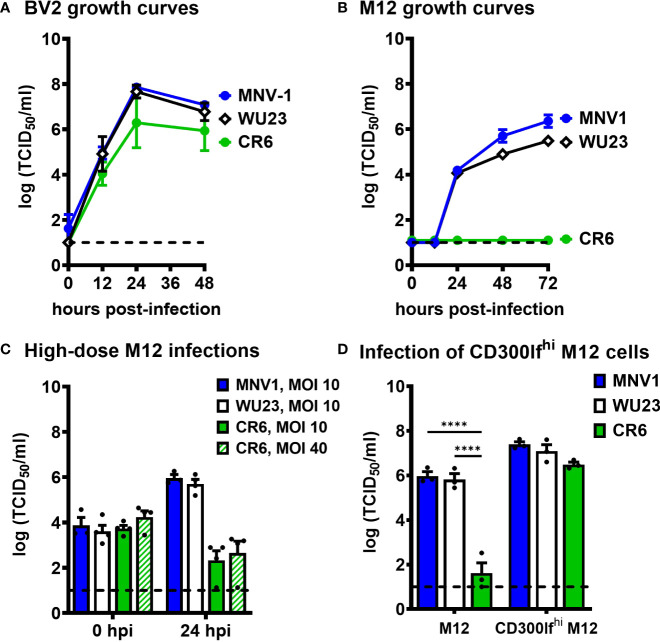
Diarrheagenic MNV1 and WU23 can infect CD300lf^lo^ cells *in vitro* while attenuated CR6 cannot. **(A, B)** Duplicate wells of BV2 cells **(A)** or M12 cells **(B)** were infected with MNV1, CR6, or WU23 at MOI 0.05. Virus titers were determined by TCID_50_ assay at the indicated timepoints on culture supernatants. *P* values were determined by ANOVA using Tukey’s multiple comparisons test. **(C)** Duplicate wells of M12 cells were infected with MNV1, CR6, or WU23 at MOI 10 or MOI 40 as indicated. Virus titers were determined by TCID_50_ assay at the indicated timepoints. **(D)** CD300lf was overexpressed in M12 cells by lentivirus transduction. Duplicate wells of M12 cells or CD300lf-overexpressing M12 cells were infected with MNV1, CR6, or WU23, at MOI 5. Virus titers were determined by TCID_50_ assay at 1 dpi. *P* values were determined using ANOVA with Sidak’s multiple comparisons test. At least three independent experiments were performed for all experiments. ****P < 0.0001.

Because virulent and attenuated MNV strains display variable cell tropism *in vivo* (5, 7, 8, 13) and VP1 is responsible for receptor binding and virus entry into permissive cells, we next questioned whether there are virus strain differences in cellular tropism *in vitro*. All three virus strains replicated comparably in BV2 cells **(** **Figure 4A** **)**, a microglial cell line susceptible to all known MNV strains (30). To confirm the finding of Graziano et al. that all the strains use the common MNV receptor CD300lf (10), we also infected *CD300lf^-/-^* BV2 cells. To our surprise, WU23 replicated modestly in these cells unlike MNV1 and CR6 **(** **Supplemental Figure 3A** **)**. Graziano et al. observed a trend towards reduced cell viability in *CD300lf^-/-^* BV2 cells infected with WU23 but not other MNV strains (10), consistent with low-level CD300lf-independent WU23 infection *in vitro*. Based on these observations, WU23 dependence on CD300lf was confirmed *in vivo* by infecting adult *Cd300lf^-/-^* and *Cd300lf^-/-^/Cd300ld^-/-^* mice, as CD300ld had previously been shown to confer susceptibility to MNV *in vitro* (11, 12). WU23 was unable to infect either strain of knockout mice **(** **Supplemental Figure 3B** **)**, so we conclude that WU23 uses the common MNV receptor *in vivo* although it is capable of modest CD300lf-independent replication *in vitro*.

Based on previous reports that MNV1 replicates in lymphocyte cell lines (7, 30), we next examined replication of MNV1, CR6, and WU23 in the M12 B cell line. Strikingly, MNV1 and WU23 replicated in M12 cells while CR6 did not **(** **Figure 4B** **)**, correlating with diarrhea severity *in vivo*. In order to gain insight into the basis for the block to CR6 infection of M12 cells, we tested whether high titers of CR6 were able to overcome the restriction, but even a dose of CR6 as high as MOI 40 failed to result in successful infection of M12 cells **(** **Figure 4C** **)**. The block to CR6 infection of M12 cells was surprising considering that MNV uses the same CD300lf receptor on BV2 and M12 cells (11). We have previously reported that CD300lf abundance is markedly different on MNV-susceptible cells *in vitro* and *in vivo*, with macrophages expressing substantially more CD300lf than lymphocytes (7). Based on this difference in receptor abundance, one explanation for our results is that CR6 affinity for CD300lf is lower than MNV1 and WU23 affinity so it is unable to infect CD300lf^lo^ cells. To test this possibility, we overexpressed CD300lf in M12 cells and confirmed successful overexpression by flow cytometry **(** **Supplemental Figure 4** **)**. CR6 infected CD300lf-overexpressing M12 cells as efficiently as MNV1 and WU23 **(** **Figure 4D** **)**, confirming that the block to CR6 infection of M12 cells is due to its inability to use low levels of the host receptor.

### Diarrheagenic MNV1 and WU23 are more responsive to environmental cues than attenuated CR6

**Figure 5:**
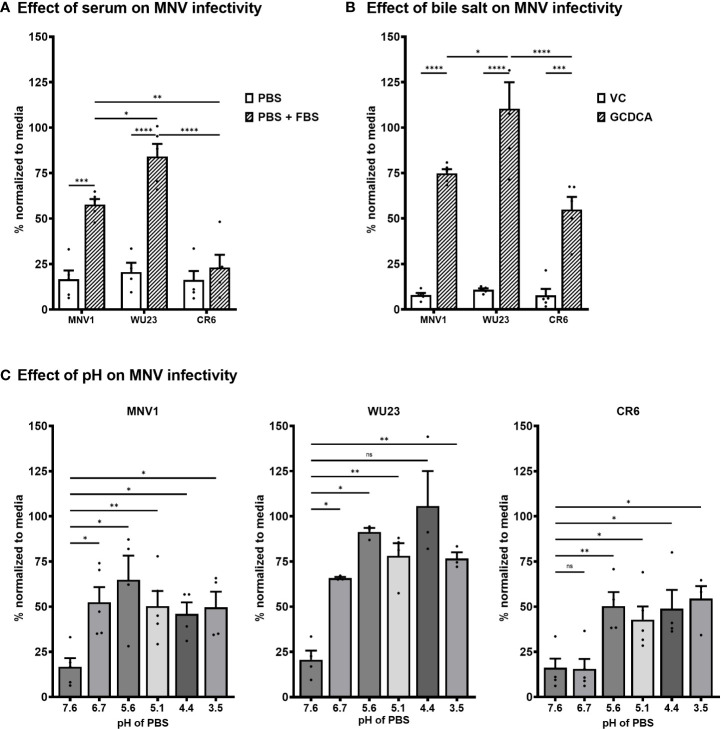
Diarrheagenic MNV1 and WU23 are enhanced by a broader profile of environmental triggers than attenuated CR6. **(A)** BV2 cells were infected with 100 TCID_50_ units per well of MNV1, WU23, or CR6 in neutral PBS, pH 7.6 or PBS supplemented with 10% FBS. Virus titers were determined by plaque assay at 2 dpi and normalized to viral titers following infection in media. *P* values were determined using ANOVA with Sidak’s multiple comparisons test. **(B)** BV2 cells were infected with 100 TCID_50_ units per well of MNV1, WU23, or CR6 250 μM GCDCA resuspended in EtOH or vehicle control (VC). Virus titers were determined by plaque assay at 2 dpi and normalized to viral titers following infection in media. *P* values were determined using ANOVA with Sidak’s multiple comparisons test. **(C)** BV2 cells were infected with 100 TCID_50_ units per well of MNV1 (left panel), WU23 (middle panel), or CR6 (right panel) in PBS at the indicated pH. Virus titers were determined by plaque assay at 2 dpi and normalized to viral titers following infection in media. *P* values were determined using ANOVA with Dunnett’s multiple comparisons test using pH 7.6 as the control group. At least three independent experiments were performed for all experiments. *P < 0.05, **P < 0.01, ***P < 0.001, ****P < 0.0001, ns, not significant.

MNV1 binding to CD300lf is influenced by a variety of environmental triggers that are present in the gastrointestinal tract, including bile salts, metal ions, and acidic pH (29, 31–34). In particular, bile salts have been demonstrated to enhance MNV1 binding to CD300lf and infectivity *in vitro*, leading to the model that environmental triggers cause noroviruses to undergo a major contraction that promotes receptor binding (29, 31–34). Based on the inability of CR6 to infect CD300lf^lo^ cells, we sought to determine whether there are virus strain-dependent differences in environmentally triggered infectivity enhancement. Consistent with published work (29), MNV1 infection of BV2 cells was inefficient when virus was applied to cells in neutral PBS but was rescued in the presence of fetal bovine serum (FBS) [**Figure 5A** **;** likely due to the presence of metal ions (29)]. FBS rescued WU23 infection comparably to MNV1 but did not appreciably enhance CR6 infection **(** **Figure 5A** **)**. Also consistent with published work (29), the bile salt glycochenodeoxycholic acid (GCDCA) enhanced MNV1 infection of BV2 cells **(** **Figure 5B** **)**. GCDCA rescued infectivity of both CR6 and WU23, although enhancement was greater for WU23 than other strains **(** **Figure 5B** **)**. Low pH also enhanced infection of each virus but there was a clear virus strain-dependent difference: MNV1 and WU23 were enhanced at pH 6.7 but CR6 required the more acidic pH of 5.6 **(** **Figure 5C** **)**. Overall, these results reveal significant differences in the ability of environmental triggers to enhance infectivity of MNV strains.

### MNV diarrheagenic potential correlates with responsiveness to environmentally triggered P domain contraction

**Figure 6:**
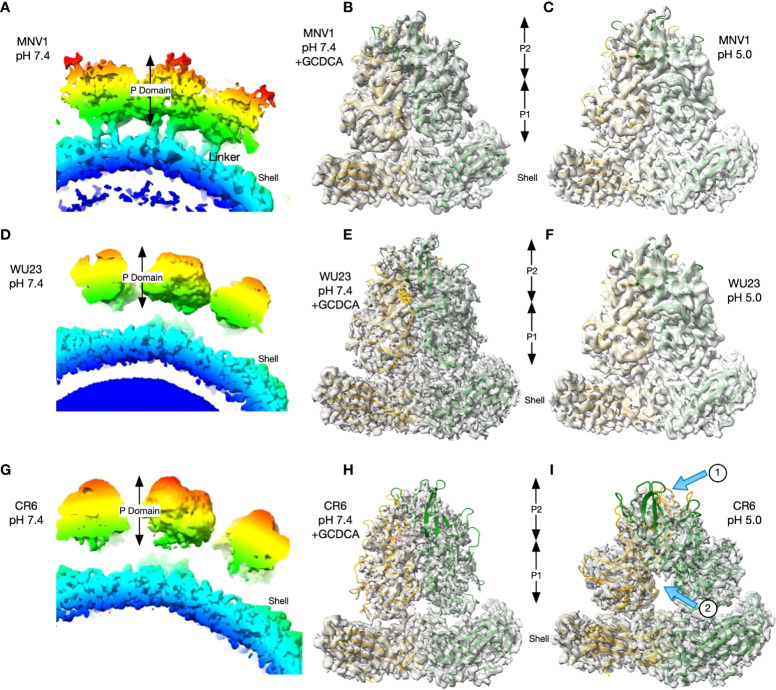
While GCDCA causes comparable contraction of virulent and attenuated MNV strains, low pH causes variable conformational changes. **(A, D, G)** Sections of the image reconstructions of all three strains at pH 7.4 without bile or metals present. Note that all three have floating P domains. The linker between the shell and the P domain is more visible in MNV1 because it is a lower resolution structure where those details are not lost. **(B, E, H)** Magnified views of the MNV P domains at pH 7.4 in the presence of GCDCA **(C, F, I)** Representative images of a magnified view of the MNV P domains at pH 5.0. In the bottom CR6 image, arrow 1 indicates the weak density of the P domains and arrow 2 indicates the splaying of P1 domains at the base that is not observed for MNV1 or WU23.

The structures of MNV1 have been determined to high resolution under a wide range of conditions (32–34). Under neutral conditions (PBS buffer, pH 7.4, without metals present) the MNV1 P domain is highly mobile and floats above the shell surface by more than 10Å **(** **Figure 6A**), with the A’B’/E’F’ loops splayed apart in an ‘open’ conformation (35). In the presence of environmental triggers found in the gut (bile salts, metal ions, or low pH), the P domain undergoes several large structural changes. The A’B’/E’F’ epitope closes, the adjacent C’D’ loop moves upward, and the P domain rotates by ~90° to contract onto the shell surface (32, 33). This closed or contracted state is optimized for receptor binding. To determine whether there are virus strain differences in these conformational changes, the structures of WU23 and CR6 were determined under a range of conditions. At pH 7.4 in the absence of metals or bile salts, the P domains of WU23 (**Figure 6D**) and CR6 (**Figure 6G**) were lifted off the shell and highly disordered as was found previously for MNV1 (**Figure 6A**). Note that these images are of a section of the whole capsid without modelling of the P domain due to the marked disorder of this region in the reconstruction. Further, the linker of the MNV1 structure is more visible than the others simply because it is a reconstruction at lower resolution where the linker can be observed. When GCDCA was added to the viruses at pH 7.4, the P domains all rotated and contracted down to the shell surface **(** **Figures 6B, E, H** **)**. While the calculated resolutions of the three reconstructions were essentially the same **(** **Table 1** **)**, the density of CR6 was markedly weaker in the outer loops and the P2 domain than observed with either MNV1 or WU23. Indeed, if the CR6 reconstructions were displayed with the same contour levels as that used for the other structures, the P domains would be entirely absent. Nevertheless, it is clear that all three strains respond to GCDCA by rotating and contracting the P domain onto the shell, consistent with infectivity data **(** **Figure 5B** **)**. The limited resolution of the outer loops of CR6 makes it impossible to know whether the details of the outer loop movement (i.e. the A’B’, E’F’, and C’D’ loops) are the same as those observed for MNV1. The P domains of all three strains also rotated and contracted onto the shell at pH 5 in the absence of metals and bile salts but there were clear virus strain-dependent differences **(** **Figures 6C, F, I** **)**: While the structures of MNV1 and WU23 at pH 5 were essentially identical to their respective structures at pH 7.4 in the presence of GCDCA, the density of the CR6 P domain was significantly weaker **(** **Figure 6I**, arrow 1). Moreover, the P domains in the dimer had a significantly different conformation with respect to each other, with the two P domains rotated about each other causing the P1 domains to splay apart at the base **(** **Figure 6I**, arrow 2).

**Table 1:** Data collection and refinement statistics.

	WU23 pH 7.4 + 10 mM GCDCA	WU23 pH 5.0	CR6 pH 7.4 +10 mM GCDCA	CR6 pH 5.0
EMD	27321	27322	27847	27849
Instrument used	Titan Krios G3i	Titan Krios G3i	Titan Krios G3i	Titan Krios G3i
# Images	13,566	8,562	9,413	8,051
# Particles Used	139,270	325,309	104,309	91,463
Software	Cryosparc v3	Cryosparc v3	Cryosparc v3	Cryosparc v3
Resolution	2.7 Å	2.9 Å	2.9Å	2.7Å

**Figure 7:**
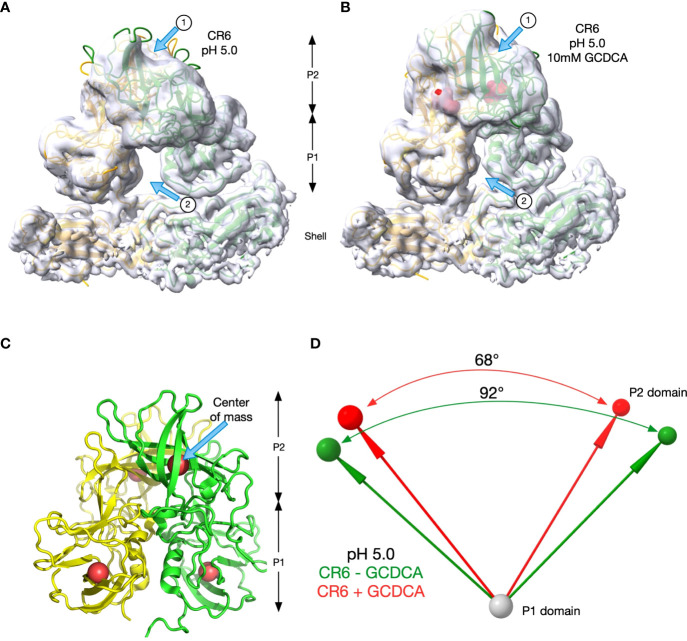
CR6 differs from the other strains at low pH. **(A, B)** The rigid body fitting results of the structure of MNV1 into the unsharpened cryo-EM maps of CR6 at pH 5 in the absence **(A)** and presence **(B)** of 10mM GCDCA. Unlike the other strains, the angles between the P domains changes at low pH in the presence and absence of GCDCA. **(C)** To measure these angles, the center of masses of the P1 and P2 domains for both subunits were calculated (red spheres). These positions were then used to calculate the vectors between the subunits as summarized in panel d. **(D)** The vectors and angles between the P domains in the CR6 structure in the presence (red) and absence (green) of 10mM GCDCA are shown.

To better visualize the apparent differences between CR6 and the diarrheagenic MNV strains, the orientations of the C/C dimers of CR6 P domains at pH 5.0 with **(** **Figure 7B** **)** and without **(** **Figure 7A** **)** GCDCA were analysed. The density for the tips of the P domains was markedly disordered in the sharpened electron micrograph (EM) density so the unsharpened maps were used in rigid body fitting of MNV1 into the densities using the program PHENIX (36). The models fit well into the densities with sufficient accuracy to estimate domain locations. The density for the P1 domains were sufficient to see isolated strands while the P2 density was more globular but the models fit well into the EM densities. As noted by the arrows, there were striking differences in the presence or absence of GCDCA. Arrow 1 notes how the P2 domains lie closer to the shell in the absence of GCDCA because the P domain is more horizonal than in the presence of GCDCA. This is also apparent by the larger gap between the P1 domains in the absence of GCDCA, indicated by Arrow 2. To quantify these observations, P1->P2 vectors were calculated between the center of mass of the P1 (residues 449-529) and P2 (residues 285-405) domains at pH 5.0 (the four red spheres in **Figure 7C** **)**. The angles between these vectors in the presence (red) and absence (green) of GCDCA were calculated by their dot products and there was a ~24° difference **(** **Figure 7D** **)**. To confirm that CR6 is indeed distinct from MNV1 and WU23 in terms of its response to low pH, the angles between the P domains dimers that lie on the icosahedral 2-fold axes (C/C dimers) were also determined for MNV1 and WU23 **(** **Table 2** **)**. For both of these virulent viruses, either low pH or bile alone was sufficient to rotate the P domains to an angle of ~70°. In all cases, the P domains contracted onto the shell surface and the density was relatively well ordered except for the outermost tips. With CR6, the P domain angles were ~70° only if GCDCA was present. Even then, the quality of the P domain density, representative of the conformational stability, was poor relative to MNV1 and WU23 **(** **Figure 6** **)**. It is possible that the amino acid differences between CR6 and the other MNV strains stabilize an intermediate in the capsid activation process at low pH where the P domains are contracted onto the shell surface but the conformational changes within the P domain have not been fully realized. Nevertheless, this is clear evidence that CR6 is less responsive to environmental cues that trigger P domain contraction, consistent with the virus strain-dependent effects of metal ions and low pH on infectivity **(** **Figures 5A, C** **)**

**Table 2:** P domain dimer angles.

Sample	Dimer angle
MNV1 pH 7.4 + GCDCA	71.8°
MNV1 pH 5.0 (no bile)	70.9°
WU23 pH 5.0 (no bile)	72.1°
CR6 pH 7.4 + GCDCA	67.7°
CR6 pH 5.0 (no bile)	92.0°
CR6 pH 5.0 + GCDCA	70.9°

### VP1-mediated regulation of B cell infection and sensitivity to environmental triggers of infectivity enhancement correlate with diarrhea

**Figure 8:**
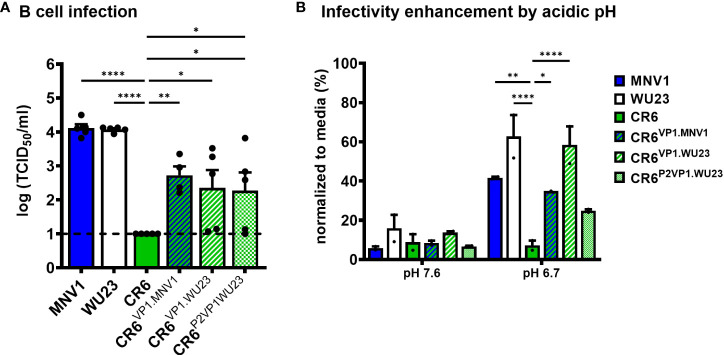
The hypervariable domain of VP1 regulates infection of CD300lf^lo^ cells and sensitivity to environmentally triggered infectivity enhancement. **(A)** Duplicate wells of M12 cells were infected with MNV1, CR6, WU23, or the indicated chimeric virus at MOI 0.05. Virus titers at 1 dpi were determined by TCID_50_ assay on culture supernatants. All infections were repeated four times. *P* values were determined using ANOVA with Dunnett’s multiple comparisons test using CR6 as the control group. **(B)** BV2 cells were infected with 100 TCID_50_ units per well of MNV1, WU23, CR6, CR6^VP1.MNV1^, CR6^VP1.WU23^, or CR6^P2VP1.WU23^ in neutral PBS (pH 7.6) or PBS at pH 6.7. Virus titers were determined by plaque assay and normalized to viral titers following infection in media. *P* values were determined using ANOVA with Dunnett’s multiple comparisons test using CR6 as the control group. The experiment was repeated two times. *P < 0.05, **P < 0.01, ****P < 0.0001.

Having determined that MNV virulence correlates with the ability to infect CD300lf^lo^ immune cells and is regulated by the P2 domain of VP1, we next sought to test directly whether VP1 regulates the ability of MNV strains to infect M12 cells. Indeed, CR6^VP1.MNV1^, CR6^VP1.WU23^, and CR6^P2VP1.WU23^ were able to infect M12 cells more efficiently than parental CR6 **(** **Figure 8A** **)**. As expected, VP1 also regulated the sensitivity of MNV strains to pH-mediated enhancement of infection **(** **Figure 8B** **)**.

## Discussion

Noroviruses are a major cause of gastroenteritis across the globe, responsible for a remarkable 685 million cases per year. Yet limitations in model systems have hindered the field’s ability to elucidate disease mechanisms. In this study, we used a recently developed small animal model of acute, self-resolving norovirus diarrhea (5, 6) to demonstrate that intestinal disease is regulated by the responsiveness of the viral capsid to environmental cues that trigger contraction of the VP1 protruding (P) domain onto the particle shell, thus enhancing receptor binding and infectivity. The capacity of a given MNV strain to undergo this contraction positively correlates with infection of cells expressing low abundance of the virus receptor CD300lf, supporting a model whereby norovirus virion contraction triggers infection of specific receptor-low cell types that are responsible for diarrhea induction.

Using the newly described neonatal mouse model of norovirus diarrhea (5, 6) coupled with the availability of genetically similar but phenotypically distinct MNV strains, we identified the hypervariable P2 domain of the VP1 capsid protein as a key virulence factor. Prior MNV studies in interferon (IFN)-deficient adult mice also identified the VP1 P2 domain, and specifically a lysine at residue 296 within this domain, as a key virulence determinant: Virulent MNV1 possesses a lysine at this position whereas attenuated strains like CR6 possess a glutamic acid and reciprocal mutations at this position are sufficient to alter virulence phenotypes (23, 25, 26). It was therefore initially surprising that residue 296 did not regulate disease in our studies. However, it is important to note that nonspecific measures of disease (e.g., weight loss, lethality, and pathology in extraintestinal sites) that most likely represent disease manifestations linked to systemic infection were used as indicators of virulence in IFN-deficient adult mice (23, 25, 26). In contrast, we measured diarrhea and colon content inconsistency as indicators of intestinal disease in neonatal mice. Further underscoring the conditional nature of the role of VP1 residue 296 in virulence, WU23 is the most virulent MNV strain characterized to date in both IFN-deficient adult mice and wild-type neonates (6) yet it possesses a glutamic acid instead of a lysine at position 296. Since acute gastroenteritis is the hallmark disease associated with human norovirus infection, virulence factors linked to MNV-induced diarrhea may have direct translatability to human noroviruses, thus highlighting the importance of using animal models that reflect the pathogenesis of human disease as closely as possible.

Having identified VP1 as a key determinant of diarrhea, we sought to elucidate the basis of its diarrheagenic activity. The P2 domain of VP1 binds the host receptor CD300lf (29, 37) so it plays a key role in viral entry and determination of cellular tropism. Consistent with this function of VP1 contributing to virulence, diarrheagenic MNV1 and WU23 infected CD300lf^lo^ M12 B cells *in vitro* while attenuated CR6 did not. CR6 could readily infect CD300lf^hi^ BV2 cells and M12 cells ectopically overexpressing CD300lf, proving that its replication defect in M12 cells is due to its inability to use low amounts of the viral receptor. Our results indicate that the VP1 P2 domain is partially responsible for this difference since CR6^VP1.MNV1^, CR6^VP1.WU23^, and CR6^P2VP1.WU23^ all displayed intermediate phenotypes in M12 replication compared to parental viruses. The intermediate nature of these phenotypes implies that a yet-to-be-identified viral factor also contributes to M12 infection. One possibility is that the minor capsid protein VP2 alters capsid conformation and is required for optimal CD300lf binding. While VP2 did not contribute to the diarrheagenic potential of MNV1 since MNV1^VP2.CR6^ caused disease comparably to parental MNV1, we were unable to determine if it contributed to the attenuation of parental CR6 since CR6^VP2.MNV1^ was replication-impaired. Thus, it remains possible that VP2 contributes to M12 cell infection and virulence. Regardless, these data illuminate a positive correlation between infection of CD300lf^lo^ M12 B cells *in vitro* and the capacity to cause diarrhea *in vivo*. Importantly, the CD300lf expression pattern *in vivo* mirrors the pattern observed in these cell lines, with high abundance on macrophages and dendritic cells and lower abundance on lymphocytes (7). Thus, it is logical to predict that lymphocyte infection contributes to MNV diarrhea. Indeed, lymphocyte-deficient *Rag1^-/-^* neonatal mice develop less severe diarrhea than wild-type mice when infected with MNV1 (5). Future studies will probe how lymphocyte infection contributes to fluid dysregulation in the intestine. Possible explanations are the secretion of immunopathologic levels of proinflammatory cytokines that act basally on the epithelium to increase permeability and thus disrupt normal fluid balance; or secretion of a viral factor that has pathologic effects on surrounding epithelial cells (e.g., a viral enterotoxin).

The interaction of monomeric CD300lf bound to the MNV P domain is a relatively weak interaction (29). Environmental factors that an enteric virus would encounter in the intestinal lumen, including bile salts, acidic pH, and metal ions, trigger the P domain to contract onto the shell of the virion and enhance its interaction with CD300lf, appearing to enable higher saturation of the virion with receptor molecules (29, 31). Our results reveal previously unappreciated virus strain-specific differences in responsiveness to these environmental cues. In particular, while the bile salt GCDCA caused comparable P domain contraction and infectivity enhancement for all three viruses examined in this study, attenuated CR6 was not as responsive to acidic conditions or metal ions as MNV1 and WU23. Indeed, CR6 appeared to only partially respond to pH alone and thus may require multiple and stronger signals for activation compared to MNV1 and WU23. This may very well explain perplexing findings in the field that, in spite of using the common host receptor CD300lf, MNV1 and WU23 primarily target intestinal immune cells in the small intestine whereas CR6 targets tuft cells in the colon (5–8, 10, 13). Moreover, since virion contraction enhances CD300lf binding and infectivity, this reduced responsiveness of CR6 to environmental triggers likely explains its inability to infect cells expressing low abundance of the viral receptor which appears to be key to disease. Noroviruses have been shown to use glycans for host cell attachment (38, 39). While there is evidence that the contraction of the P domain enhances interaction with the MNV entry receptor CD300lf, contraction of the P domain could also facilitate increased interaction with attachment factors (40).

MNV binding to both CD300lf and bile salt can be markedly diminished by as few as two mutations in the P domain (29), providing support that relatively few differences in this region between virus strains could be sufficient to dramatically alter infectivity. Because MNV1, WU23, and CR6 are highly genetically related viruses and there is precedence for a single residue regulating MNV virulence [e.g., VP1 residue 296 in IFN-deficient adult mice (23, 25, 26)], we attempted to determine whether any individual P2 domain residue controlled virulence in neonates. There are six P2 residues shared between diarrheagenic MNV1 and WU23 but different for attenuated CR6 (S299A, I301T, A305V, K351R, A365V, and V374A) which were individually mutated in the parental CR6 backbone and tested *in vivo* but none of these changes conferred increased virulence **(** **Supplemental Figure 5** **)**. These findings suggest that multiple P2 residues combinatorially determine diarrheagenic potential. Alternatively, it is possible that distinct P2 residues determine virulence for MNV1 and WU23. Finally, although the P2 domain of WU23 conferred increased virulence to CR6, CR6^P2VP1.WU23^ was not quite as virulent as CR6^VP1.WU23^ or parental WU23, suggesting that residues in the P1 or S domains of VP1 also regulate virulence. Future studies will test combinatorial mutations to determine precise residues across VP1 associated with virulence.

Altogether, our results reveal that, although there is a common MNV receptor (10), diarrheagenic viruses display increased responsiveness of virion contraction to environmental cues compared to attenuated viruses, expanding their tropism to include receptor-low cellular targets. Considering that diarrheagenic MNV1 and WU23 are highly genetically related to attenuated CR6, it is evident that subtle genetic differences among norovirus strains can dramatically influence cellular tropism and virulence. When considering the much greater genetic heterogeneity that exists among human norovirus strains, it is fascinating to speculate about the translatability of these findings. Underscoring this point, while there are 34 amino acid differences across the VP1 protein of MNV1 and CR6, individual genogroup II, genotype 4 (GII.4) human norovirus VP1 proteins can differ by nearly 60 amino acids and the sequence divergence between genotypes and genogroups is far greater (41, 42). There are conflicting reports in the human norovirus field about cellular tropism, with certain studies revealing infection of epithelial cells and others revealing infection of immune cells (17–22, 30). This contradiction could be explained by variable responsiveness to environmental cues among human norovirus strains. Consistent with this possibility, some human noroviruses require bile to infect intestinal organoids *in vitro* while other strains do not (43). Moreover, a GII.4 human norovirus has recently been reported to undergo a similar contraction to MNV in response to metal ions (24), demonstrating that this is a common phenomenon impacting norovirus pathogenesis. It is thus possible that differential responsiveness to environmental cues leads to variable cellular tropism and levels of virulence among human norovirus strains. Overall, this body of work illustrates the utility of having a tractable small animal model of norovirus infection to elucidate the relationship between the basic biology of noroviruses and intestinal disease.

## Methods

### Cells and viruses

BV2, *CD300lf^-/-^* BV2, and HEK293T cells were maintained in Dulbecco’s modified Eagle medium supplemented with 10% fetal bovine serum, 100 U penicillin/mL, and 100 μg/mL streptomycin. M12 cells were maintained in Roswell Park Memorial Institute 1640 media (RPMI) with 100 U penicillin/mL, 100 μg/mL streptomycin, and 10% fetal bovine serum. For structural studies, RAW264.7 cells were used to generate virus as described below and previously (31). Cells tested negative for mycoplasma. MNV-1.CW3 (GenBank accession number KC782764, referred to as MNV1), MNV-CR6 (GenBank accession number JQ237823.1, referred to as CR6), and MNV-WU23 (GenBank accession number EU004668.1 but altered as described previously (6), referred to as WU23) infectious clones were used to generate virus stocks. Infectious clones for all chimeric viruses between MNV1 and CR6 were generated as described previously (23). The infectious clone for MNV1^VP1.K296E^ was generated as described previously (25). For CR6 and WU23 chimeric viruses, primers were designed using the NEBuilder HiFi DNA Assembly primer design tool. These primers were utilized to amplify two fragments of DNA: the backbone virus strain on an infectious clone plasmid without the gene of interest and the gene of interest from the other virus strain. These fragments then were combined using NEBuilder HiFi DNA Assembly and subsequently transformed into *E.coli*. Colonies were screened and the correct sequence was confirmed by sequencing. For all CR6 mutant viruses, specific mutations were introduced into the CR6 infectious clone plasmids with mutagenic primers using an Agilent QuikChange Lightning site-directed mutagenesis kit according to the manufacturer’s protocol. Colonies were screened and the correct mutation was confirmed by sequencing. To generate virus stocks, 10^6^ HEK293T cells were transfected with 5 µg of endotoxin-free infectious clone using Lipofectamine 3000. Cells were frozen after 30 h, lysed by freeze-thaw, and lysates applied to BV2 cells. BV2 lysates were frozen when cultures displayed 90% cytopathic effect and then freeze-thawed twice, cell debris removed by low-speed centrifugation, followed by purification of virus through a 25% sucrose cushion. Virus was dissolved in phosphate-buffered saline (PBS). Virus stocks were titered by a standard TCID_50_ assay as described previously (9). Stocks were sequenced to confirm no mutations arose during generation. Initial WU23 experiments were performed with original virus (kindly provided Dr. Craig Wilen, Yale University). Once the infectious clone plasmid was available, virus stock for WU23 was made up in the same way as other stocks. Both methods of producing WU23 stock produced similar *in vivo* results (6). A mock inoculum stock was prepared in the same manner using BV2 lysate from uninfected cultures.

### Mice

Specific pathogen-free (SPF) mice used in this study were bred and housed in animal facilities at the University of Florida and Washington University School of Medicine. All animal experiments were performed in strict accordance with federal and university guidelines and approved by the Institutional Animal Care and Use Committee at the University of Florida (study numbers 20190632 and 202200000065) and Washington University School of Medicine (study number 20190162). The conditions in animal rooms used in this study fall within the standards set by the “Guide for the Care and Use of Laboratory Animals.” Additionally, all mice were bred under MNV-free conditions. For adult mouse experiments, age- and sex-matched 6-12 week-old C57BL/6J (Jackson no. 000664, referred to as B6), C57BL/6J-*CD300lf^-/-^* (10, 11) and C57BL/6J-*CD300lf^-/-^/CD300ld* ^-/-^ (kindly provided by Craig Wilen, Yale University) mice were used. Adult mice were inoculated perorally (p.o.) with 10^7^ TCID_50_ units of the indicated virus strain or mock inoculum. For neonatal mouse experiments, 3d old male and female BALB/c (Charles River no. 028) or B6 (Jackson Laboratories no. 000664) pups were used. Neonatal mice were inoculated with the indicated MNV strain or mock inoculum using oral gavage (5) or intragastric inoculation. For the latter procedure, 40 μL of inoculum was injected directly into the stomach (which can be visualized through the skin in the upper left quadrant) using a 30-gauge needle and 1 mL BD syringe. These two inoculation methods produce similar *in vivo* results (6). Pups were weighed at 2 dpi and the abdomen of each pup was palpated to induce defecation. Fecal condition was assessed based on color and consistency according to a 5-point scale: 1, firm, orange, does not smear; 2, pasty, orange or mixed color, does not smear; 3, orange or yellow, semi-liquid and smears; 4, yellow, liquid, and smears; 5, green-yellow, non-viscous liquid. Any pup that did not defecate was excluded from the experiment since feces could not be scored. Any pup that scored a 3-5 was considered to have diarrhea for the purpose of calculating incidence. For certain experiments, the consistency of colon contents was also scored after sacrificing the mouse utilizing the scoring system for fecal scores.

### Virus titration assays

For cell culture infections and virus stock production, virus titers were determined by a standard TCID_50_ assay as described previously (9) with minor modifications. Eight replicates each of multiple dilutions per sample were applied to BV2 cells. Cytopathic effect was scored at 3 dpi. For virus titer determination in animal tissues, plaque assays were performed. Harvested tissues were immediately placed in tubes with 1.0 mm zirconia/silica beads and 1 mL of complete DMEM and flash frozen. Samples were subsequently thawed and homogenized by bead beating. Multiple dilutions of tissue samples were applied to BV2 cells. Plates were then incubated at room temperature with rocking. After 1 h, plates were overlaid with 1.5% SeaPlaque Agarose and modified eagle medium (MEM) supplemented with 10% fetal bovine serum, 100 U penicillin/mL, and 100 μg/mL streptomycin. Plates were then incubated for 2 days at 37°C. Cells were stained with 1.5% SeaKem Agarose in phosphate-buffered saline (PBS) with neutral red, and plaques counted 3-4 h later.

### *In vitro* infections

BV2 or M12 cells were inoculated with MNV1, WU23, CR6, or the indicated chimeric virus at the indicated MOI and incubated for 1 h with rocking at room temperature. Cells were then washed once with PBS to remove unbound virus, fresh media added, and incubated at 37°C. Supernatants were collected from duplicate wells per condition at the noted timepoints. Virus titers were determined by TCID_50_ assay. Three independent growth curves were performed.

### CD300lf overexpression

The gene expressing CD300lf (Genscript OMu11937) was cloned into pGenlenti by Genscript. Lentivirus was produced in HEK293T cells by co-transfection of this plasmid with the packaging plasmid psPAX2 (Addgene 12260) and envelope plasmid pMD2.G (Addgene 12259). The lentivirus was then transduced into M12 cells and 0.8 µg/mL puromycin added to the culture supernatant to select for transduced cells. After selection, cells were maintained in 0.5 µg/mL puromycin. Overexpression was confirmed by flow cytometry by staining with 0.1µg of a polyclonal anti-mouse antibody to CD300lf (R&D Systems, AF2788) followed by staining with anti-goat IgG secondary antibody conjugated to AlexaFluor 594 (Invitrogen A32758). M12 cells or M12 cells overexpressing CD300lf were inoculated with MNV1, WU23, or CR6 at MOI 5 and incubated for 1 h with rocking at room temperature. Cells were then washed with PBS to remove unbound virus, fresh media added, and incubated at 37°C. Supernatants were collected from duplicate wells per condition at 1 dpi. Virus titers were determined by TCID_50_ assay.

### Structural studies

WU23 and CR6 were produced and purified as previously described (31). In brief, when RAW264.7 cells reached a density of ~0.5-1.0x10^6^ cells/mL, ~6-8 L of cells were harvested by centrifuging 4,000g for 10 min. The cells were suspended in ~400 mL in HEPES (AH) media, placed into a 4 L flask and ~1x10^9^ particle forming units (PFU) of WU23 added. The suspension was slowly shaken for 1 h at room temperature, ~800 mL of fresh AH media was added, and then transferred to a 37° incubator without CO_2_ and shaken at ~70 rpm in the dark for 24-32 h. The infected cell suspension was centrifuged for 30 min at 5,000g and the supernatant was collected. To the supernatant, dry NaCl and PEG 8,000 was added to yield 0.3M and 10%, respectively. The solution was then mixed at 4°C overnight. The solution was then centrifuged for 30 min at 10,000g and the pellet was resuspended in 50-80 mL of PBS. After incubation at 4°C for several hours, the debris was removed by centrifugation at 10,000g for 30min. To the supernatant, glycerol was added to a final concentration of 10% (v/v) as a cryoprotectant, divided into 1.5 mL aliquots, and stored at -80°C. Immediately prior to the cryo-EM experiments, 50-100 mL of this material was thawed and centrifuged at 45,000 rpm for 2 h. The pellets were resuspended in less than a total of 3 mL of PBS and allowed to incubate for several hours at 4°C. Debris was removed by centrifugation and the supernatant was then layered onto Beckman SW41 tubes containing 7.5-45% linear sucrose gradients using PBS as buffer. After centrifugation for 1.5-2.0 h at 35,000 rpm at 4°C, MNV forms a band ~2/3 of the way down the tube. The virus was collected *via* puncturing the side of the tube with a syringe. The pooled bands were split into two and dialyzed overnight at 4°C against the final buffer used in the experiment (i.e., PBS at pH 7.4 or citrate/phosphate at pH 5.0). The following day, the virus samples were placed in the 10 mL tubes for a Beckman 70.1 Ti rotor and centrifuged at 48,000 rpm for 1.5 h. The pellets were then resuspended in ~100 µL of the dialysis buffer and frozen for cryo-EM. Purified WU23 at pH 5 and 7.4 were at concentrations of ~1 mg/mL. In addition to the pH 7.4 vitrification, the same sample with 10 mM GCDCA (final concentration) added was vitrified. The virus was vitrified as previously described (44) on carbon holey film (R2x1 Quantifoil^®^; Micro Tools GmbH, Jena, Germany) grids. Briefly, grids were cleaned in Gatan 950 Solarus plasma cleaner for 40 s in hydrogen-oxygen gas mixture. 4 µL of the virus solutions were applied to the holey films, blotted with filter paper, and plunged into liquid ethane. The EM-GP2^®^ (Leica) automated plunger was used for vitrification. The grids were screened for ice and sample quality and imaged in a Titan Krios G3i (Thermo-Fisher) microscope. The microscope was equipped with a Gatan K3 BioQuantum (Ametek, Inc.) and operated at 300 keV. A slit width of 20 eV was used for data collection. Images were acquired in EPU (Thermo Fisher) using fast acquisition mode with beam-image shift used for hole centering instead of stage movement. The direct detector camera (K3, Ametek) operated in super-resolution counted mode, images were recorded with overall electron dose of 48 electrons/Å^2^; the defocus range was -1.5 to -2.5 µm. The data collection statistics are summarized in **Table 1**. For all three data sets, particles were picked using the Cryosparc3 (45) template picker and culled using 2D classification. The initial model was generated using the *ab-initio* routine in Cryosparc2 using icosahedral symmetry. This initial model was used as an initial volume in the non-uniform refinement algorithm and after several cycles yielded a cryo-EM density with an effective resolution of the MNV structures summarized in **Table 1** according to 0.143 Gold Standard Fourier shell correlation function (FSC) criterion.

Since the structure of CR6 at pH 5.0 without bile differed from MNV1 and WU23 at pH 5.0, additional analyses were performed. The EM density for the P domains was weak for CR6 at pH 5.0 and slightly better in the presence of 10mM GCDCA. Therefore, using the unsharpened maps from Cryosparc2 refinement, the two copies of the MNV1 P domains were fitted into the density using the rigid body routines in PHENIX. **Figure 7** shows the two sets of maps and models of the C/C dimers. The differences were obvious in the maps (arrows in **Figures 7A**, **B**) and those differences were reflected in the fitted models. While CR6, pH 5.0, in the presence of GCDCA looked essentially the same as MNV1 and WU23 at pH 5.0 (no bile) or at pH 7.4 plus bile, the domains in CR6 at pH 5.0 were decidedly tilted differently with respect to each other. To verify and quantify these differences, vectors were calculated for each of the P domains in the C/C dimers. The base of each vector was the center of mass of the P1 domain (residues 449-529) and the head was the center of mass of the P2 domain (285–405). Those vectors are shown in **Figure 7D**. From the dot product of the vectors, the angles between the P domains in the dimers were calculated. **Table 2** summarizes all measured angles in the other MNV structures.

### Bile acid and pH enhancement assays

BV2 cells were plated and allowed to adhere overnight. MNV1, CR6, WU23, or the indicated chimeric virus was diluted to 100 TCID_50_ per well in either DMEM, PBS, PBS supplemented with 250 μM glycochenodeoxycholic acid (GCDCA), PBS supplemented with ethanol added as a vehicle control, PBS supplemented with 10% FBS, or PBS with sufficient phosphoric acid to achieve the indicated acidic pHs. Calcium- and magnesium-free PBS was used throughout this protocol. After the cells were washed with PBS, virus was added for 1 h at room temperature with rocking. After absorption, inoculum was aspirated, and plates were overlaid with 1.5% SeaPlaque Agarose and modified eagle medium (MEM) supplemented with 10% fetal bovine serum, 100 U penicillin/mL, and 100 μg/mL streptomycin. Plates were then incubated for 2 d at 37°C. Cells were stained with 1.5% SeaKem Agarose in PBS with neutral red and plaques counted 3-4 h later.

### Statistical analysis

All data were analyzed with GraphPad Prism software. *P* values were determined using one-way or two-way ANOVA with corrections for multiple comparisons for all experiments except fecal scores and incidence of diarrhea. For fecal scores and incidence of diarrhea, *P* values were determined using Kruskal Wallis test with corrections for multiple comparisons. Error bars denote standard errors of mean in all figures. P values less than 0.05 are indicated by one asterisk, P values less than 0.01 are indicated by two asterisks, P values less than 0.001 are indicated by three asterisks, and P values less than 0.0001 are indicated by four asterisks.

## Data availability statement

The original contributions presented in the study are included in the article/**Supplementary Material**. Further inquiries can be directed to the corresponding author.

## Ethics statement

The animal study was reviewed and approved by University of Florida (study numbers 20190632 and 202200000065) and Washington University School of Medicine (study number 20190162).

## Author contributions

EWH, AMP, TJS, and SMK conceived the experiments and wrote the manuscript. Everyone else generated data.

## Funding

This work was supported by the National Institutes of Health (NIH) grants R01AI162970 (SK), R01AI141478 (SK, MB), and R01AI123144 (SK). EH was supported by NIH F30AI154834. AP was supported by NIH T32AI007110. MP was supported by NIH T90DE021990. EK was supported by NSF Graduate Research Fellowship DGE-1745038/DGE-2139839 and NIH F31AI167499. JA was supported by a McKnight Doctoral Fellowship. MB was supported by R01AI139314 and R01AI127552 (MB) and the Pew Biomedical Scholars Program of the Pew Charitable Trusts. TS was supported by R01AI141465.

## Acknowledgments

We thank Dr. Craig Wilen for generously providing original WU23 virus stock and the *CD300lf^-/-^/CD300ld^-/-^* knockout mouse line. We also acknowledge the support of the Sealy Center for Structural Biology at the University of Texas Medical Branch.

## Conflict of interest

The authors declare that the research was conducted in the absence of any commercial or financial relationships that could be construed as a potential conflict of interest.

## Publisher’s note

All claims expressed in this article are solely those of the authors and do not necessarily represent those of their affiliated organizations, or those of the publisher, the editors and the reviewers. Any product that may be evaluated in this article, or claim that may be made by its manufacturer, is not guaranteed or endorsed by the publisher.
