# Supplementary material for: Environmentally-triggered contraction of the norovirus virion determines diarrheagenic potential
Source: Front Immunol. 2022 Nov 1;13:1043746. doi: 10.3389/fimmu.2022.1043746 (PMC9664153; doi:10.3389/fimmu.2022.1043746)
Supplement: Supplementary file 1 [file DataSheet_1.pdf]

Supplemental Figure 1. Colon content consistency scores reveal that VP1 is the major virulence factor.

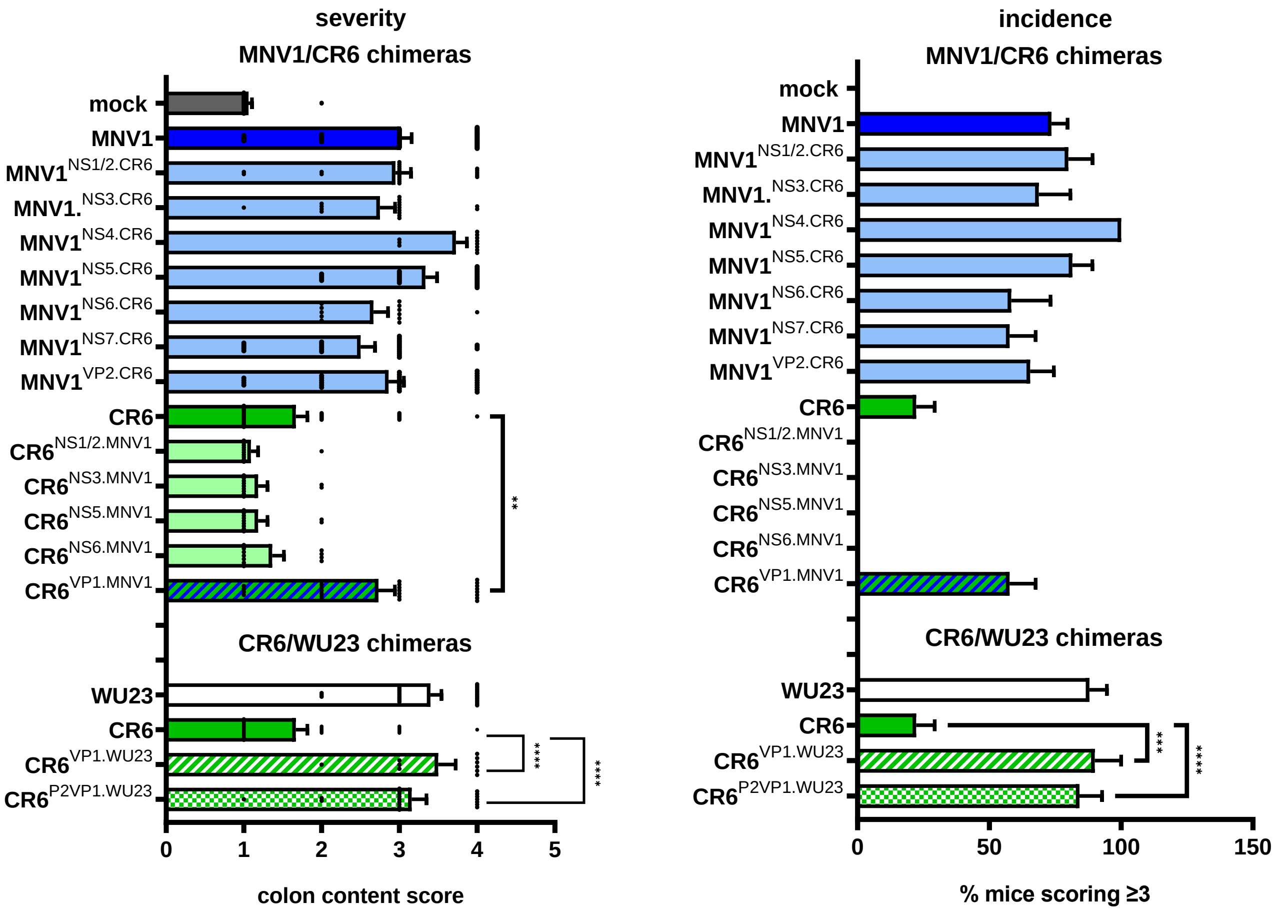

Supplemental Figure 1. Groups of P3 BALB/c neonatal mice were inoculated with 10^7 $TCID_{50}$ units of mock inoculum, WU23, MNV1, CR6, or the indicated chimeric virus. At 2 dpi, pups were sacrificed, and colon content consistency scored (left graph). The proportion of mice scoring a 3 or above is presented as incidence of diarrhea (right graph). At least 10 mice from at least two independent litters were analyzed. Error bars denote standard errors of mean in all figures. *P* values were determined using Kruskal-Wallis test with Dunn's multiple comparisons test for both the MNV1/CR6 chimeras and the CR6/WU23 chimeras by comparing each chimeric virus to its respective parental virus.

Supplemental Figure 2: The VP1 296 residue is not responsible for virulence in neonatal mice.

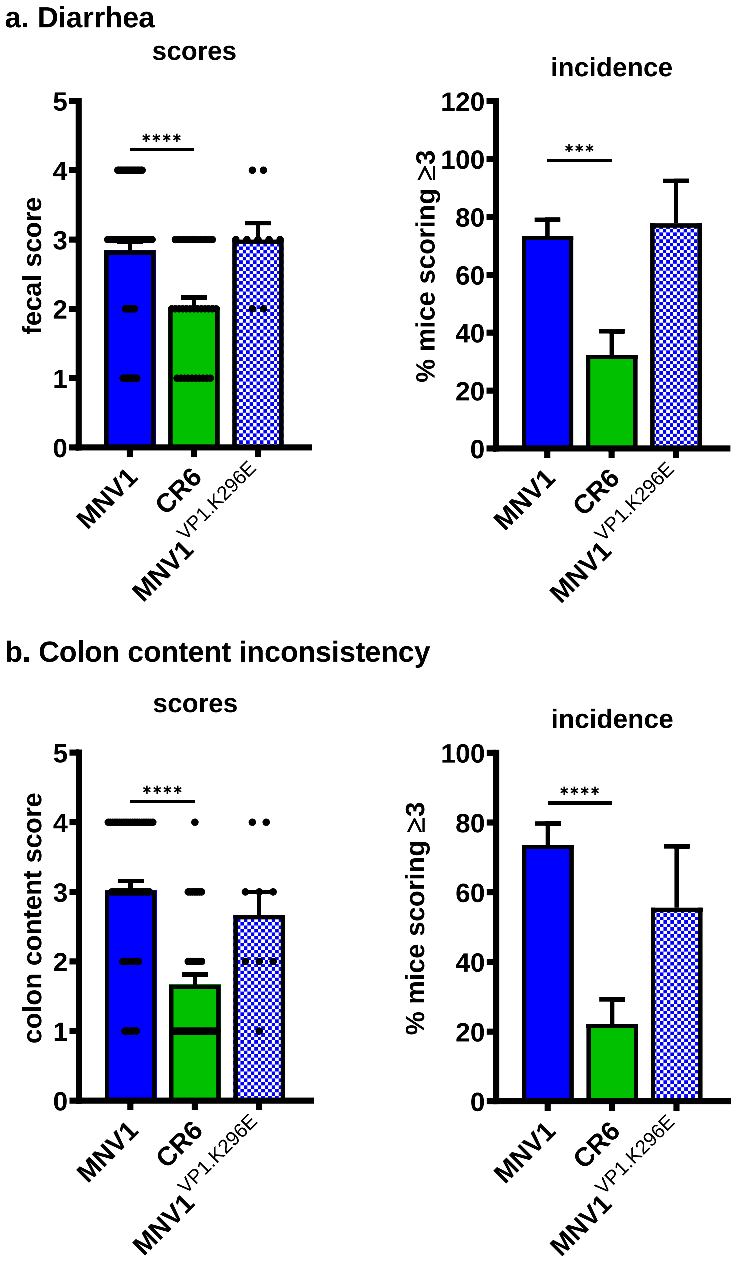

Supplemental Figure 2. The VP1 296 residue is not responsible for virulence in neonatal mice. Groups of P3 BALB/c pups were inoculated with 10^7 $TCID_{50}$ units of MNV1, CR6, or MNV1$^{VP1.K296E}$. At least nine mice from at least two independent litters were analyzed for each condition. **a)** At 2 dpi, fecal consistency was determined by palpating their abdomens (left graph). The proportion of mice scoring a 3 or above is presented as incidence of diarrhea (right graph). **b)** At 2 dpi, the consistency of colon contents was determined (left graph). The proportion of mice with colon contents scoring a 3 or above is presented as incidence of diarrhea for colon content scores (right graph). Error bars denote standard errors of mean in all figures. *P* values were determined using Kruskal-Wallis test with Dunn's multiple comparisons test using MNV1 as the control group.

Figure 3. CD300lf is required for WU23 replication in vivo but not in vitro.

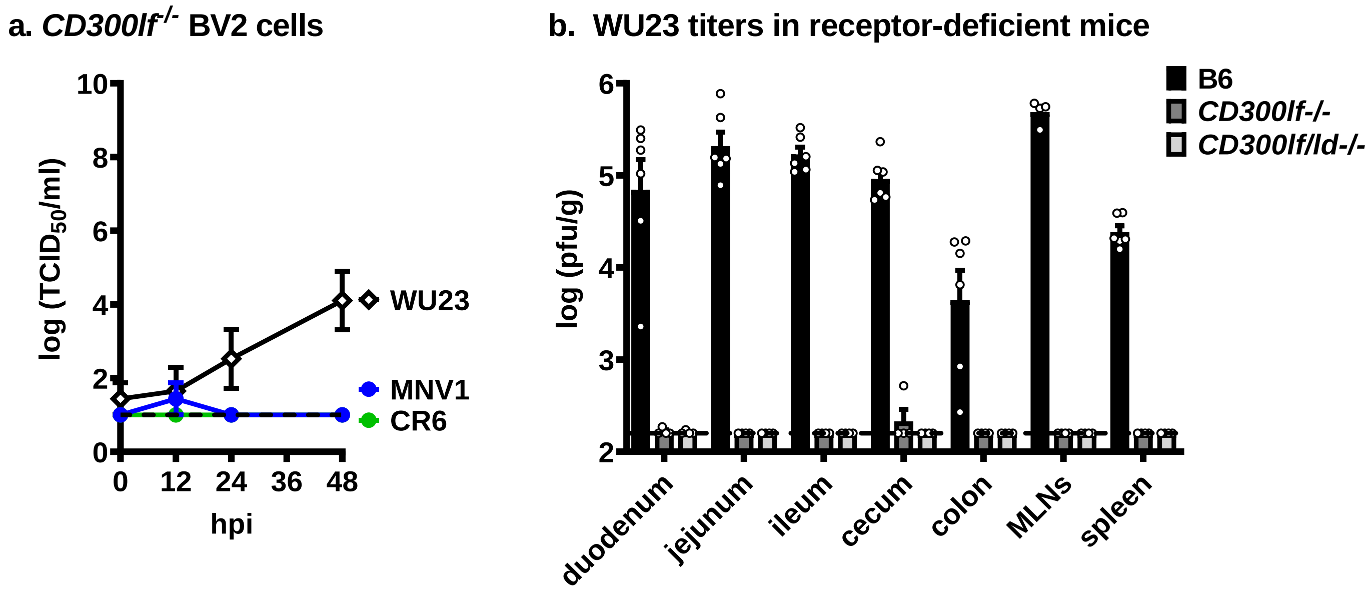

Supplemental Figure 3. CD300lf is required for WU23 replication in vivo but not in vitro. a) Duplicate wells of *CD300lf*$^{-/-}$ BV2 cells were infected with MNV1, WU23, or CR6 at MOI 0.05. Supernatants were collected at the indicated timepoints and virus titers were determined by $TCID_{50}$ assay. Three independent experiments were performed. **b)** Groups of adult B6 (*n = 6*), *Cd300lf*$^{-/-}$ (*n = 4*), and *Cd300lf/ld*$^{-/-}$ (*n = 4*) mice were infected p.o. with 10^7 $TCID_{50}$ units of WU23. At 1 dpi, viral titers were determined in the indicated segments of the intestinal tract, mesenteric lymph nodes, and spleen by plaque assay.

Supplemental Figure 4. Confirmation of CD300lf overexpression in M12 cells.

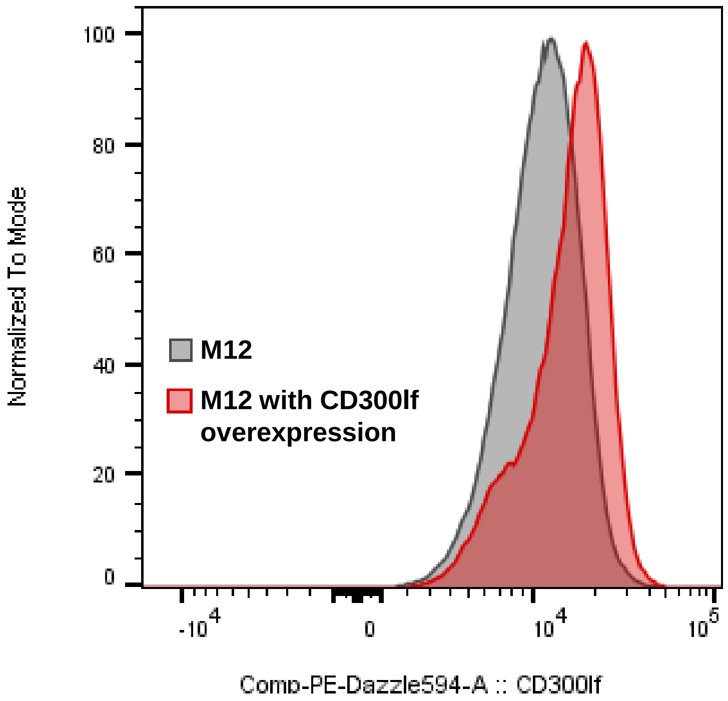

Supplemental Figure 4. Confirmation of CD300lf overexpression in M12 cells. CD300lf was overexpressed in M12 cells by lentivirus transduction. M12 cells and CD300lf-overexpressing M12 cells were stained with a polyclonal goat antibody for CD300lf followed by a secondary antibody conjugated to Alexa Fluor 594. Cells were analyzed by flow cytometry for expression levels of CD300lf in two independent experiments.

Supplemental Figure 5: No individual P2 residue is responsible for virulence in neonatal mice.

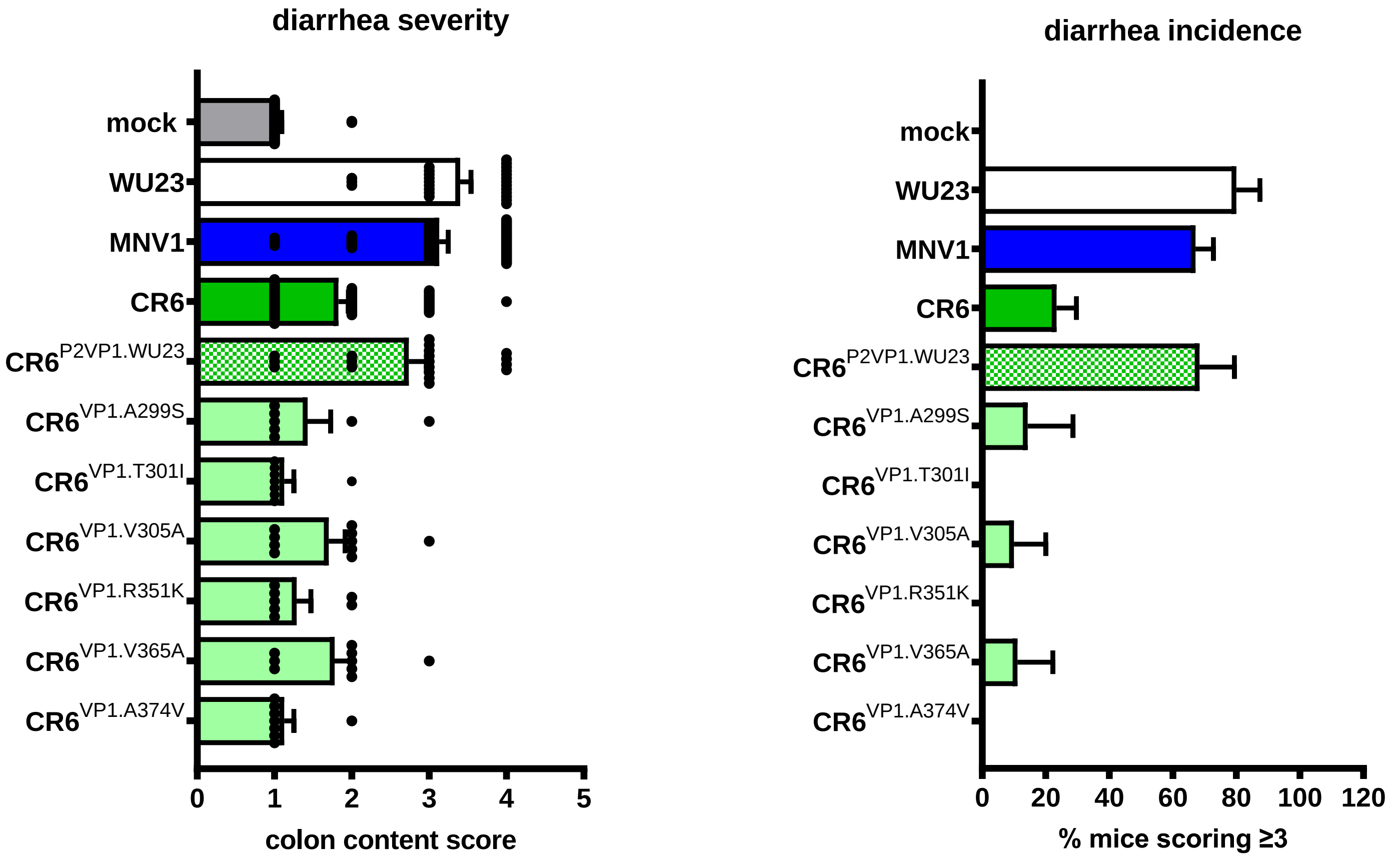

Supplemental Figure 5. No individual P2 residue is responsible for virulence in neonatal mice. Groups of P3 BALB/c pups were inoculated with 10^7 $TCID_{50}$ units of mock inoculum, WU23, MNV1, CR6, or the indicated mutant virus. At 2 dpi, pups were sacrificed, and colon content consistency scored (left graph). The proportion of mice scoring a 3 or above is presented as incidence of diarrhea (right graph). At least seven mice were analyzed. Error bars denote standard errors of mean in all figures. *P* values were determined using Kruskal-Wallis test with Dunn's multiple comparisons test by comparing CR6 to each mutant virus.
